# Improving diagnostic efficacy of primary prostate cancer with combined ^99m^Tc-PSMA SPECT/CT and multiparametric-MRI and quantitative parameters

**DOI:** 10.3389/fonc.2023.1193370

**Published:** 2023-09-11

**Authors:** Yu Zhang, Yuanying Shi, Liefu Ye, Tao Li, Yongbao Wei, Zhiyi Lin, Wenxin Chen

**Affiliations:** ^1^ Department of Nuclear Medicine, Shengli Clinical Medical College of Fujian Medical University, Fuzhou, China; ^2^ Department of Nuclear Medicine, Fujian Provincial Hospital, Fuzhou, China; ^3^ Department of Nuclear Medicine, Fujian Research Institute of Nuclear Medicine, Fuzhou, China; ^4^ Department of Urology, Shengli Clinical Medical College of Fujian Medical University, Fuzhou, China; ^5^ Department of Urology, Fujian Provincial Hospital, Fuzhou, China

**Keywords:** prostate-specific membrane antigen, magnetic resonance imaging, prostate cancer, single-photon emission computed tomography, technetium radioisotopes

## Abstract

**Purpose:**

This prospective study aimed to evaluate the difference between ^99m^Tc-PSMA single-photon emission computed tomography (SPECT)/CT and multiparametric magnetic resonance imaging (mpMRI) in the detection of primary prostate cancer (PCa).

**Materials and methods:**

Fifty-six men with suspected PCa between October 2019 and November 2022 were prospectively enrolled in this study. The median age of the patients was 70 years (range, 29-87 years). Patients were divided into high-(Gleason score>7, n=31), medium- (Gleason score=7, n=6) and low-risk groups (Gleason score < 7, n=6). All patients underwent ^99m^Tc-PSMA SPECT/CT and mpMRI at an average interval of 3 days (range, 1-7 days). The maximum standardized uptake value (SUV_max_), the minimum apparent diffusion coefficient (ADC_min_), and their ratio (SUV_max_/ADC_min_) were used as imaging parameters to distinguish benign from malignant prostatic lesions.

**Results:**

Of the 56 patients, 12 were pathologically diagnosed with a benign disease, and 44 were diagnosed with PCa. ^99m^Tc-PSMA SPECT/CT and mpMRI showed no significant difference in the detection of primary PCa (kappa =0.401, *P*=0.002), with sensitivities of 97.7% (43/44) and 90.9% (40/44), specificities of 75.0% (9/12) and 75.0% (9/12), and AUC of 97.4% and 95.1%, respectively. The AUC of SUV_max_/ADC_min_ was better than those of SUV_max_ or ADC_min_ alone. When SUV_max_/ADC_min_ in the prostatic lesion was >7.0×10^3^, the lesion was more likely to be malignant. When SUV_max_/ADC_min_ in the prostatic lesion is >27.0×10^3^, the PCa patient may have lymph node and bone metastases. SUV_max_ was positively correlated with the Gleason score (*r*=0.61, P=0.008), whereas ADC_min_ was negatively correlated with the Gleason score (*r*=-0.35, *P*=0.023). SUV_max_/ADC_min_ was positively correlated with the Gleason score (*r*=0.59, *P*=0.023). SUV_max_/ADC_min_ was the main predictor of the high-risk group, with an optimal cut-off value of 15.0×10^3^.

**Conclusions:**

The combination of ^99m^Tc-PSMA SPECT/CT and mpMRI can improve the diagnostic efficacy for PCa compared with either modality alone; SUV_max_/ADC_min_ is a valuable differential diagnostic imaging parameter.

## Introduction

Prostate cancer (PCa) is one of the most common malignancies in men (1). Early diagnosis and accurate grading of PCa are of great significance for formulating therapeutic strategies and improving prognosis (2). Multiparametric magnetic resonance imaging (mpMRI) is a well-established tool for the appraisal of primary PCa and has shown high affectability (3, 4). Prostate biopsy remains the gold standard for PCa diagnosis. In addition to providing evidence for diagnosis, the pathological results can also provide the classification and grouping information of PCa. Ultrasound-guided puncture biopsy still has a high false-negative rate of 20-25%, and there are complications such as bleeding, infection, pain, and urinary retention (3). Therefore, it is important to explore a noninvasive preoperative diagnosis method for PCa to improve puncture accuracy and avoid unnecessary biopsy. Current strategies used to locally stage PCa and recognize the exact location of disease foci depend on the results of systematic or targeted biopsies and mpMRI. However, mpMRI has limited specificity (4–6). Although targeted mpMRI biopsies have significantly improved the identification of clinical PCa, there is still over a 30% chance of missing primary PCa in men (7). Therefore, additional complementary methods are required to better characterize and identify primary PCa.

Prostate-specific membrane antigen (PSMA) is a type II transmembrane protein that is overexpressed on the surface of 90% of PCa cells. Its expression positively correlates with the degree of malignancy (8). Published studies have demonstrated the superiority of ^68^Ga/^18^F-PSMA PET/CT or PET/MR in the detection of primary PCa. However, PET/CT or PET/MR is not widely available in less developed countries, and far fewer institutions have PET/CT or PET/MR devices than SPECT/CT devices. The limited production of ^68^Ga from ^68^Ge-^68^Ga generator and ^18^F from cyclotron, combined with the relatively short half-life of ^68^Ga (67.71 min) and ^18^F (109.8min), results in the need for multiple rounds of production per day to maintain patient use, limiting the number of patient tests per day. Although clinical SPECT system sensitivity and resolution are not as good as those of PET, the recent combination of SPECT and CT and the ability to quantify tissue radioactivity concentration in absolute units have resulted in a significant improvement in imaging quality. ^99m^Tc, available from ^99^Mo-^99m^Tc generators, is a nuclide routinely used in SPECT imaging, has good physical properties (half-life is 361.2 min), and is inexpensive and widely available. Thus, ^99m^Tc-based PSMA ligands are a cost-effective clinical alternative. Our previous study showed that ^99m^Tc-labelled PSMA molecular probe (^99m^Tc-HYNIC-Glu-Urea-A, herein referred to as ^99m^Tc-PSMA) single-photon emission computed tomography (SPECT)/CT can display bone metastases of PCa with high sensitivity and specificity (9), with only a small amount of radiation uptake in the intestinal tract and no significant radiation uptake in other major organs (10). However, to our knowledge, ^99m^Tc-PSMA SPECT/CT has rarely been reported for the diagnosis of primary PCa. In recent years, with the development of imaging technology, mpMRI including functional sequences such as diffusion weighted imaging(DWI) had been widely used in the diagnosis and preoperative localization of PCa (5). The ADC_min_ from DWI reflects the degree of diffusion of water molecules in the tumor tissue. SUV_max_ represents PSMA expression associated with the biological characteristics of tumors. Our study aimed to evaluate the difference between ^99m^Tc-PSMA SPECT/CT and mpMRI for the detection of primary PCa.

## Materials and methods

### Ethical approval

This study was approved by the ethics committee of Fujian Provincial Hospital (reference number, K2019-10-017) and conducted in compliance with the principles of the Declaration of Helsinki. Furthermore, informed consent was obtained from all participants and/or their legal guardians.

### Sample size calculation

We conducted a prospective head-to-head observational study to analyze the diagnostic efficacy between ^99m^Tc-PSMA SPECT/CT and mpMRI in treatment-naive PCa. In this study, the sensitivity and specificity of ^99m^Tc-PSMA SPECT/CT and mpMRI in the diagnosis of PCa were assumed to be greater than 50% (H_0 = _50%). Referring to similar published literature on ^68^Ga-PSMA PET/CT and mpMRI (11), a sensitivity and specificity value of 80% was assumed. PASS 11 software (Power Analysis and Sample Size, NCSS, LLC) was used to estimate the required sample size. Assuming α=0.05 (unilateral), β=0.1, and a 1:1 ratio between the groups, the calculations indicated that at least 46 patients needed to be included in the study. Consequently, 56 individuals were enrolled in this study.

### Patient selection

Fifty-six men were enrolled in this study between October 2019 and November 2022. The inclusion criteria were as follows (2): ① digital rectal examination touching the prostate nodules; ② transrectal ultrasound suspected PCa; ③ PSA>10 ng/mL or progressive PSA increase (12); ④ no treatment administered before the scan; and ⑤ complete medical records, control data, and clinical follow-up results. The exclusion criteria were as follows: ① the presence of severe syndromes that were difficult to manage; ② active or upcoming participation in other clinical drug trials; ③ lack of regular review or follow-up results; ④ a second primary tumor, and ⑤ inability to obtain relevant contrast imaging and clinical data. All eligible patients underwent ^99m^Tc-PSMA SPECT/CT and mpMRI at an average interval of 3 days (1–7 days). None of the patients received antineoplastic therapy between the two scans. After both scans were completed, a transrectal needle prostate biopsy was performed. The patient characteristics are presented in **Table 1**.

**Table 1:** Patient characteristics.

Patient characteristic	Value
No. of patients	56
Age (years), median (IQR)	70 (29-87)
serum PSA(ng/mL), median (IQR)	14.8 (5.1-710.0)
PI-RADS score, n (%)	
1-2	8 (14.3%)
3	5 (8.9%)
4-5	43 (76.8%)
Pathological features of the specimen	
benign nodules, n (%)	12 (21.4%)
adenocarcinoma, n (%)	43 (76.8%)
neuroendocrine carcinoma, n (%)	1 (1.8%)
Gleason score	
<7 (low risk), n (%)	6 (14.0%)
=7 (intermediate risk), n (%)	6 (14.0%)
>7 (high risk), n (%)	31 (72.0%)
IUSP GG	
1-3 (low-grade), n (%)	12 (28.0%)
≥4 (high-grade), n (%)	31 (72.0%)
Prostatectomy	
Yes, n (%)	23 (41.1%)
No, n (%)	33 (58.9%)

IQR, interquartile range; IUSP GG, International Society of Urological Pathology Grade Group; PSA, prostate specific antigen; PI-RADS, prostate imaging reporting and data system.

### ^99m^Tc-PSMA SPECT/CT acquisition protocol

The PSMA lyophilized kit (HYNIC-PSMA) (patent number,Zl202010878750.4) was provided by the Shanghai Engineering Research Centre of Molecular Imaging Probes. The synthesis procedure has been reported previously (9). The radiochemical purity was>95%. All patients were injected intravenously with a dose of 0.74 GBq (20 mCi) ^99m^Tc-PSMA. Whole-body planar imaging and regional (neck-pelvic) SPECT/CT were performed 2 h after injection using a Discovery NM/CT 670Pro (GE, USA) with low-energy, high-resolution collimators. The image acquisition protocol was as follows: planar imaging: peak energy 140 keV (^99m^Tc) and scan velocity 15 cm/min in a 256×1025 matrix. Regional SPECT/CT: camera matrix size 128×128, zoom 1.0, rotation 360°, and 30 s/frame for 60 frames. Low-dose CT (130 keV; 60 mA) was used.

### mpMRI acquisition protocol

mpMRI was performed with a high-field system (Magnetom Prisma 3.0T, Siemens, The Germany) using a standardized protocol with pelvic external phased-array coils. The sequences included: transverse T1-weighted imaging (T1WI) (repetition time [TR]=500ms, echo time [TE]=12ms, field-of-view [FOV]=20 cm×20 cm, matrix=320×256); T2-weighted imaging (T2WI) (TR=5800ms, TE=106ms, FOV=20 cm×20 cm, matrix=320×256); fat-suppression spectral presaturation attenuated inversion recovery-T2WI (TR=5800ms, TE=97ms, FOV=20 cm×20 cm, matrix=320×240), and diffusion weighted imaging (DWI) (TR=5100ms, TE=64ms, FOV=20 cm×20 cm, matrix=114×114, b=50s/mm^2^, 600 s/mm^2^, 1500 s/mm^2^, 3000 s/mm^2^). The section thickness of each sequence was 3.5 mm.

### Image analysis

^99m^Tc-PSMA SPECT/CT and mpMRI images were independently read by two nuclear medicine physicians and two radiologists, respectively. The readers were blinded to the mpMRI and ^99m^Tc-PSMA SPECT/CT clinical reports and other readers’ findings. ^99m^Tc-PSMA SPECT/CT and mpMRI were performed on a workstation (Xeleris, General Electric, Waukesha, WI) and (syngo.via, Siemens Healthineers, respectively). The locations of lesions on mpMRI and ^99m^Tc-PSMA SPECT/CT images were compared, and lesions with the same locations on the two scans were selected as the primary lesion to extract parameters for analysis.

### Diagnostic criteria for primary PCa

On mpMRI, combined with the reconstructed apparent diffusion coefficient (ADC) images, a lesion with a prostate imaging reporting and data system (PI-RADS) score > 3 was considered a positive lesion (PCa) (13). The lesions’ region of interest (ROI) was delineated, and the lowest ADC (ADC_min_) was calculated. On SPECT/CT, areas with higher imaging agent uptake than normal prostate tissue after excluding physiological uptake were considered positive lesions (PCa). For imaging-based quantification analysis, Q.Metrix software (Q.Metrix GE Healthcare) was used (14). Acquisition information, including camera sensitivity, activities in full and empty syringes, administration time, and scan time, was input into the system. The volume of interest (VOI) was delineated, and the NM was 0.4. The calculated maximum standardized uptake value (SUV) voxel volume was 3.2×10^-3^ mL. VOI-related quantitative parameters were automatically generated, and SUV_max_ was used for quantitative analysis.

### Diagnostic criteria for PCa metastases

On mpMRI, ①lymph node metastases: round, short-axis diameter>8 mm, uneven signals in lymph nodes on T2WI, irregular boundaries, and evident enhancement on dynamic contrast-enhanced (DCE) (15); ② bone metastases: low signal intensity on T1WI and T2WI, limited diffusion on DWI, and early enhancement after contrast agent injection on DEC (16, 17). On SPECT/CT, ①lymph node and bone metastases: uptake than normal tissue(lesion SUV_max_≥liver SUV_max_) after excluding lacrimal glands, salivary glands, kidneys, bladder and intestines physiological uptake. The SUV_max_ of all focal SPECT-positive sites was determined based on the ROI basis (17). In the quantification analysis, the size of each SPECT-positive bone and lymph node correlated with the SUV_max_.

### Validation of findings

Prostate needle biopsies were performed in all participants. We used a protocol for transperineal MRI/PSMA-ultrasound fusion targeted and systematic biopsy. In brief, the image-guide (cognitive guidance, MRI/US and PSMA/US) technique was used. Targeted and systematic biopsies were performed in the same session. The number of biopsy cores was as follows: 3-4 cores for targeted biopsy and 10-12 cores for systematic biopsy. If the biopsy results were positive, patients with surgical indications underwent radical prostatectomy, and the pathological results were based on the gross specimen. For patients without surgical indications, pathological results were based on biopsy results. If the needle biopsy results are negative and the clinical symptoms are highly indicative of PCa, the patient’s serum PSA value and imaging (mpMRI, ^99m^Tc-PSMA SPECT/CT) should be followed up for 3-6 months. If the disease does not progress, PCa could be excluded. If the disease progresses, an additional needle biopsy should be performed (12). Not all bone and lymph node lesions showed positive pathological results. Thus, the validated method reported in previous studies was used (9, 17). All patients were followed up for at least 6 months (or until death). Serum PSA levels were reviewed every 3 months for all patients. The subsequent therapeutic schedule options depended on the patient’s condition, including radical prostatectomy, local radiation therapy, and chemotherapy. Future imaging modalities were selected according to their respective clinical needs and were not bound by a specific protocol. Patients who met at least one of the following conditions were metastases: ① Response to therapy (hormone therapy and radiation) and subsequent serum PSA decline were confirmed by follow-up examination (MRI, CT, PET, etc.); ② two or more imaging examinations recommended metastases, and ③ PSA≥100 ng/mL, suggesting distant metastases (18).

### Statistical analysis

Data analysis was performed using SPSS 19.0 software (statistical product and service solutions, Chicago, Illinois). McNemar’s test was used to compare the cancer detection concordance rates between ^99m^Tc-PSMA SPECT/CT and mpMRI. The Mann-Whitney *U* test was used to compare the differences in quantitative diagnostic parameters among the different groups. Receiver operating characteristic (ROC) analysis was performed to evaluate the sensitivity, specificity, area under the ROC curve (AUC), and a cut-off value of each parameter. The Kruskal-Wallis test was used to compare the differences in quantitative diagnostic parameters among different tumor size groups. The correlation between the Gleason Score and SUV_max_, ADC_min_, and SUV_max_/ADC_min_ was evaluated using Spearman correlation analysis. Logistic regression analysis was used to calculate predictors of the Gleason score. *P*<0.05 was considered statistically significant.

## Results

### Overall results

**Figure 1:**
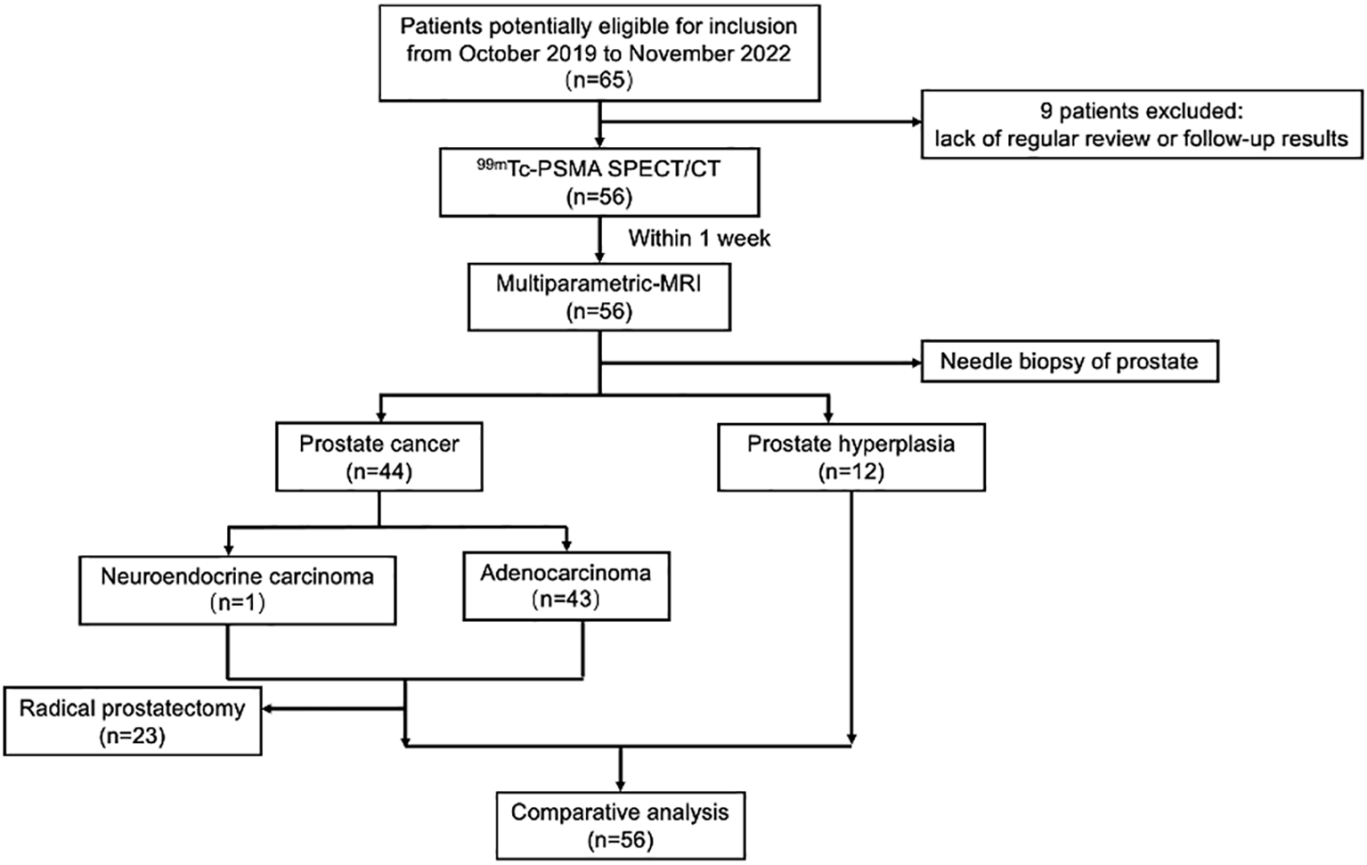
Flowchart of participant selection in the study. ^99m^Tc-PSMA SPECT/CT, ^99m^Tc-labelled prostate-specific membrane antigen molecular probe single photon emission computed tomography; MRI, magnetic resonance imaging.

Among the 56 participants, 44 (78.5%) were diagnosed with PCa, and 12 (21.5%) with prostate hyperplasia (BPH). A flowchart illustrating the participant inclusion procedure is shown in **Figure 1**. Among the 44 patients with PCa, one (2%) had neuroendocrine carcinoma, and 43 (98%) had adenocarcinoma. The surgical indications were judged by the urological surgeon according to the clinical status of the patient (2).The 23 patients with PCa diagnosed by puncture underwent robot-assisted laparoscopic radical prostatectomy (RP); postoperative pathology results were consistent with those of puncture in 11 (11/23,47.8%) patients. 12 (12/23,52.2%) patients with PCa experienced pathological upgrading. The Gleason score of patients who underwent surgery was based on the surgical specimen, and the Gleason score of patients who did not undergo surgery was based on the puncture specimen. Among 44 patients with PCa, 23 (52.3%) had metastases.

### Sensitivity and specificity analyses

**Figure 2:**
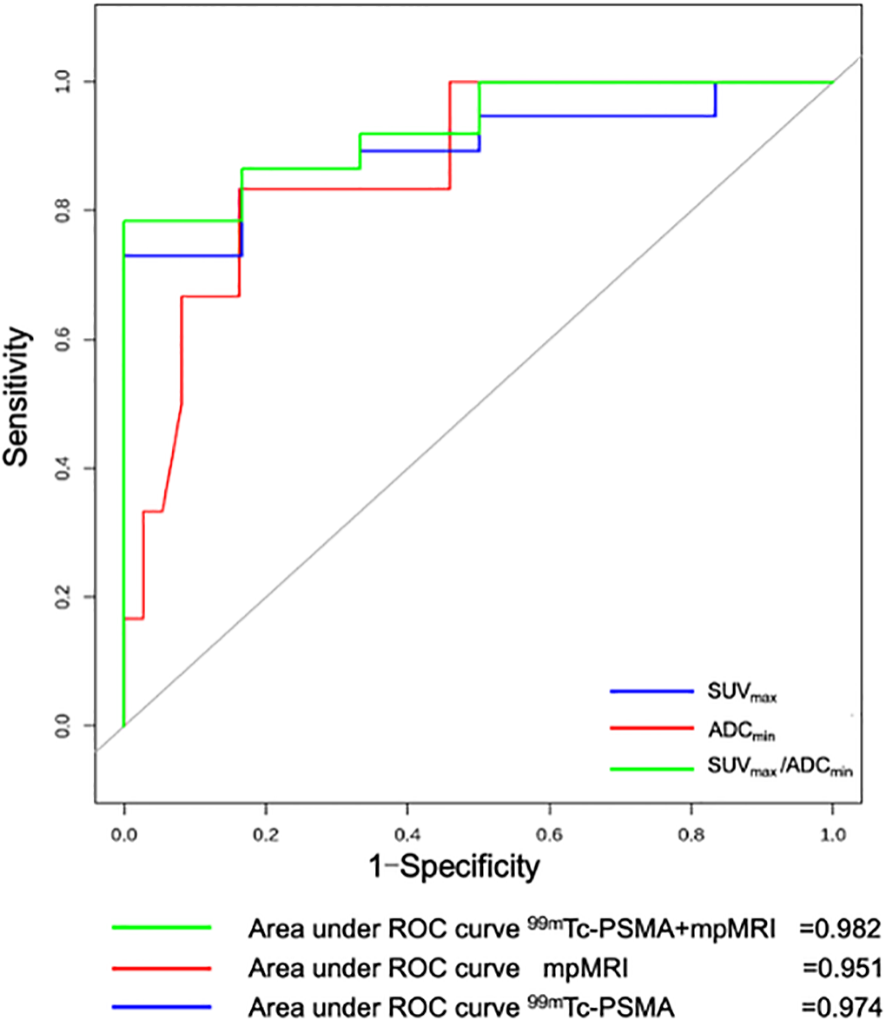
Receiver operating curve (ROC) for ^99m^Tc-PSMA SPECT/CT, mpMRI, and ^99m^Tc-PSMA SPECT/CT+ mpMRI for detection of primary prostate cancer (n=56). ^99m^Tc-PSMA SPECT/CT, ^99m^Tc-labelled prostate-specific membrane antigen molecular probe single photon emission computed tomography; mpMRI, multiparametric magnetic resonance imaging; SUV_max_, maximum standardized uptake value; ADC_min_, the minimum apparent diffusion coefficient.

For all 56 patients, the sensitivity of ^99m^Tc-PSMA SPECT/CT and mpMRI in detecting primary PCa was 97.7% (43/44) and 90.9% (40/44), respectively; the difference was not statistically significant (χ^2 = ^0.102, *P*=0.749). Their specificity was 75.0% (9/12) and 75.0% (9/12), respectively, with no statistically significant difference (χ^2 = ^1.333, *P*=0.248) (**Table 2**). ROC curve analysis revealed an accuracy, as measured by AUC, of 97.4% (*95% CI.* 93.7%-100.0%) for ^99m^Tc-PSMA, 95.1% (95% CI. 88.8%-100.0%) for mpMRI, and 98.2% (*95% CI.* 95.2%-100.0%) for ^99m^Tc-PSMA+mpMRI (**Figure 2**).

**Table 2:** ^99m^Tc-PSMA SPECT/CT and mpMRI in the diagnosis of primary prostate cancer.

Pathological diagnosis	^99m^Tc-PSMA		mpMRI	
	Positive	Negative	Positive	Negative
Positive (n=44)	43	1	40	4
Negative (n=12)	3	9	3	9
Total	46	10	43	13
PPV	0.935 (95% CI. 0.863-1.000)		0.930 (95% CI. 0.854-0.100)	
NPV	0.900 (95% CI. 0.714-1.000)		0.692 (95% CI. 0.414-0.943)	

CI, confidence interval; PPV, positive predictive value; NPV, negative predictive value; ^99m^Tc-PSMA SPECT/CT, ^99m^Tc-labelled prostate-specific membrane antigen molecular probe single photon emission computed tomography; mpMRI, multiparametric magnetic resonance imaging.

### Differences in quantitative parameters among different groups

**Figure 3:**
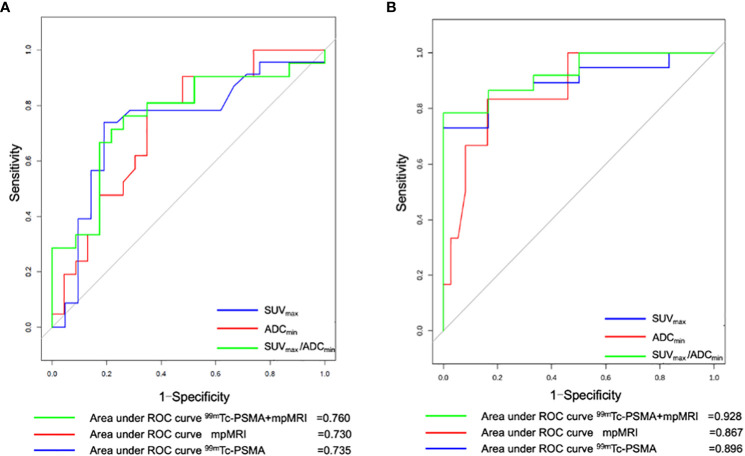
Receiver operating curve (ROC) for **(A)** ^99m^Tc-PSMA SPECT/CT, mpMRI, and ^99m^Tc-PSMA SPECT/CT+ mpMRI for detecting prostate cancer with metastases (n=44) and for **(B)** ^99m^Tc-PSMA SPECT/CT, mpMRI and ^99m^Tc-PSMA SPECT/CT+ mpMRI for detection of prostate cancer with Gleason score ≥7 (n=43). ^99m^Tc-PSMA SPECT/CT, ^99m^Tc-labelled prostate-specific membrane antigen molecular probe single photon emission computed tomography; mpMRI, multiparametric magnetic resonance imaging; SUV_max_, maximum standardized uptake value; ADC_min_, the minimum apparent diffusion coefficient.

The Mann-Whitney *U* test was used to compare differences in quantitative diagnostic parameters among the different groups (**Table 2**). The SUV_max_/ADC_min_ of the PCa group was significantly higher than that of the BPH group, and the SUV_max_/ADC_min_ of the subgroup with metastases was higher than that of the subgroup without metastasis (**Table 2**). In ^99m^Tc-PSMA SPECT/CT combined with mpMRI, when the cut-off value for SUV_max_/ADC_min_ was set at 7.0×10^3^, the sensitivity and specificity of SUV_max_/ADC_min_ in PCa were 93.2% (95%*CI.*85.7%-100.0%) and 100.0% (95%*CI.*100.0%-100.0%), respectively, with a Youden index of 0.932 and an AUC of 0.982 (95% CI. 95.2%-100.0%) (**Figure 3**). When the cut-off value for SUV_max_/ADC_min_ was set at 27.0×10^3^, the sensitivity and specificity of SUV_max_/ADC_min_ in PCa with metastases was 76.2% (95%*CI.*58.0%-99.4%) and 73.9% (95%*CI.*56.0%-91.9%), respectively, with a Youden index=0.501 and AUC=0.760 (*95% CI.* 61.0%-91.0%) (**Figure 3A**).

### Difference between tumor size and quantitative parameters

**Figure 4:**
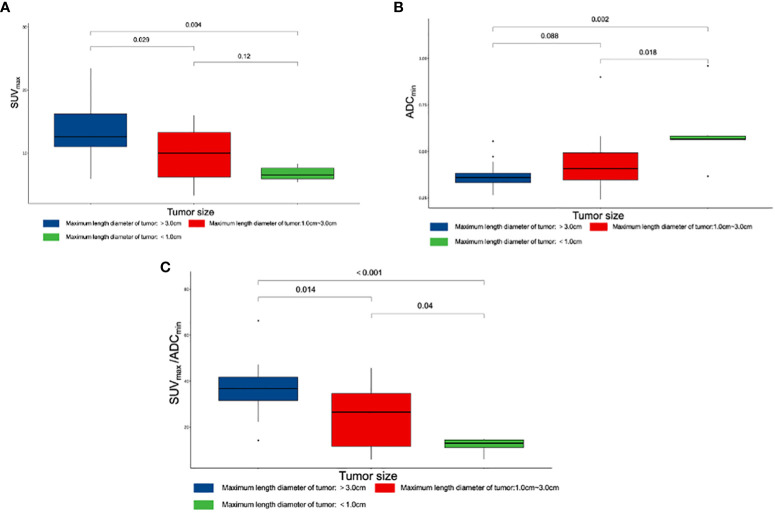
Box plot of different parameters and tumor size. **(A)** Differences among SUV_max_ and tumor size. **(B)** Differences among ADC_min_ and tumor size. **(C)** Differences among SUV_max_/ADC_min_ and tumor size. SUV_max_, maximum standardized uptake value; ADC_min_, the minimum apparent diffusion coefficient.

The 44 prostatic lesions detected were grouped according to their maximum tumor diameter: G1 (7/44, maximum diameter < 1.0 cm), G2 (23/44, maximum diameter: 1.0 cm-3.0 cm), and G3 (14/44, maximum diameter > 3.0 cm). The Kruska–Wallis test was used to compare the differences in quantitative diagnostic parameters among different tumor size groups. There were differences in SUV_max_/ADC_min_ among the tumor size groups; the larger the tumor size, the larger the SUV_max_/ADC_min_ value (**Figure 4**).

### Relationship between Gleason score and quantitative parameters

**Figure 5:**
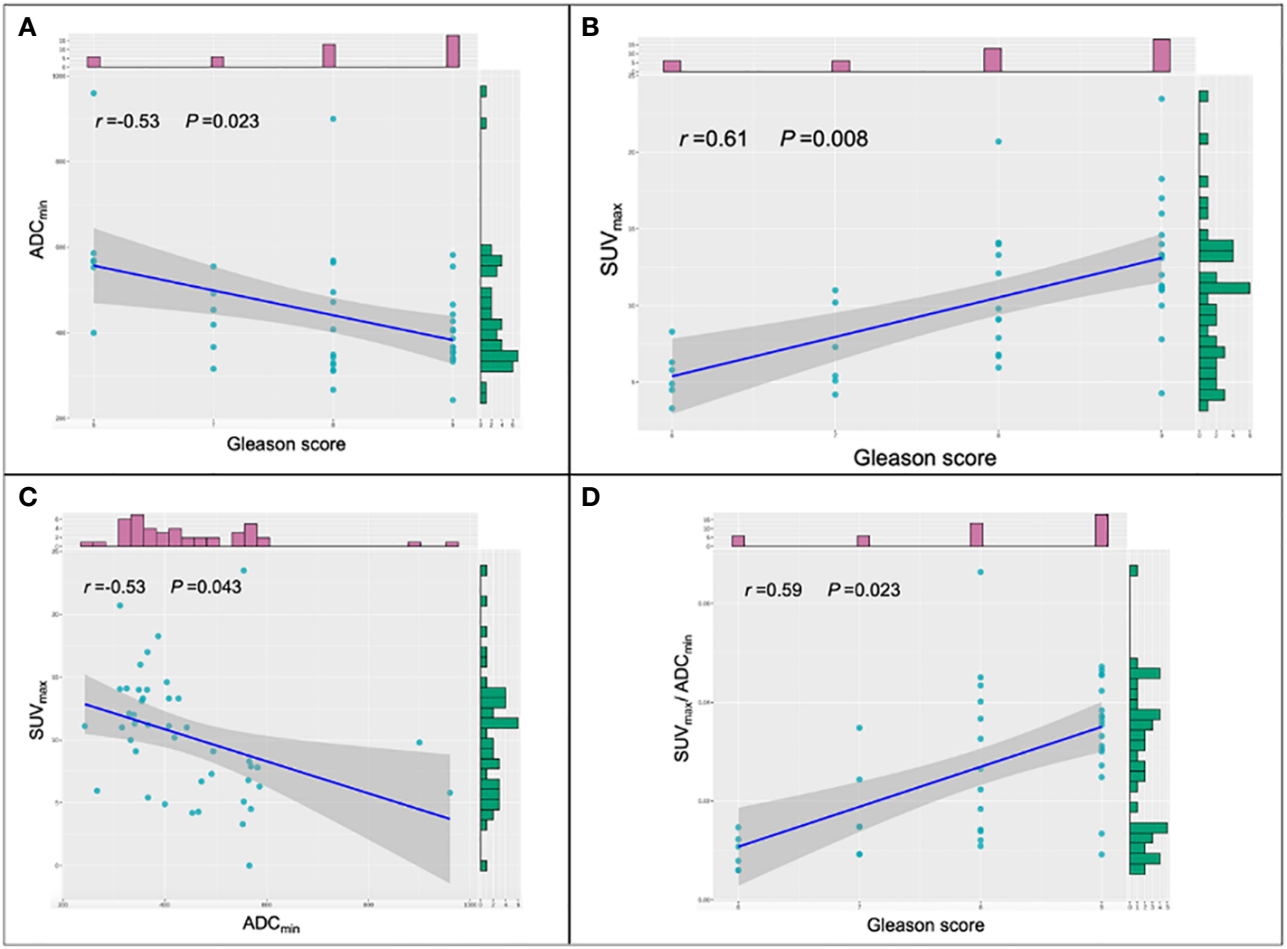
Scatter plots of the different parameters and Gleason score. **(A)** Correlations among ADC_min_ and Gleason score in prostate cancer (PCa) lesions. **(B)** Correlations among SUV_max_ and Gleason score in PCa lesions. **(C)** Correlations among SUV_max_ and ADC_min_ in PCa lesions. **(D)** Correlations among SUV_max_/ADC_min_ and Gleason score in PCa lesions. SUV_max_, maximum standardized uptake value; ADC_min_, the minimum apparent diffusion coefficient.

Gleason scoring is unsuitable for treating neuroendocrine PCa (19). Therefore, 43 patients with PCa were enrolled in this cohort study. Spearman correlation analysis was used, and the results revealed that ADC_min_ showed a weak negative correlation with Gleason score (*r*=-0.35, *P*=0.023), whereas SUV_max_ (*r*=0.61, *P*=0.008) and SUV_max_/ADC_min_ (*r*=0.59, *P*=0.023) showed a moderate positive correlation with Gleason score (**Figure 5**). Based on the Gleason score, the patients were divided into high-(Gleason score>7, n=31), medium- (Gleason score=7a, n=4;Gleason score=7b, n=2) and low-risk groups (Gleason score < 7, n=6). According to the Mann-Whitney *U* test, there were statistical differences in SUV_max_, ADC_min,_ and SUV_max_/ADC_min_ between the high-, medium- and low-risk groups (all *P* < 0.05) (**Table 3**). With the presence of a high-risk group (yes=1, no=0) as the dependent variable, and age, serum PSA level, and SUV_max_/ADC_min_ as the independent variables, logistic regression analysis showed that SUV_max_/ADC_min_ was independently correlated with the presence of a high-risk group; for every 1×10^3^ increase in SUV_max_/ADC_min_, the detection rate of the high-risk group increased by 55.7% (*OR*=1.557, *P*=0.042). When the cut-off value for SUV_max_/ADC_min_ was set at 15.0×10^3^, the sensitivity and specificity of SUV_max_/ADC_min_ in the high-risk PCa group were 78.4% (95%*CI.*65.1%-91.6%) and 100.0% (95%*CI.*100.0%-100.0%), respectively, with a Youden index of 0.784 and AUC of 0.928 (*95% CI.* 84.0%-100.0%) (**Figure 3B**) (**Table 4**).

**Table 3:** Difference between the three diagnostic parameters among different groups.

Group (No. of patients)	^99m^Tc-PSMA	mpMRI	^99m^Tc-PSMA+ mpMRI
	SUV_max_	ADC_min_	SUVmax/ADC_min_
Prostate hyperplasia (n=12)	0.00 (0.00-0.54) ^△^	0.69 (0.57-0.81)×10^-3□^	0.00 (0.00-6.51)×10^3▽^
Prostate cancer (n=44)	10.60 (0.00-23.48)	0.41 (0.24-0.96)×10^-3^	26.87 (0.00-663.50)×10^3^
Gleason score<7(n=6)	4.90 (0.00-8.30) ^*^	0.57 (0.40-0.96)×10^-3#^	8.22 (0.00-14.66)×10^3★^
Gleason score=7(n=6)	6.360 (4.20-11.00)	0.44 (0.32-0.56)×10^-3^	14.80 (9.19-34.82)×10^3^
Gleason score>7(n=31)	12.00 (4.28-23.48)	0.37 (0.24-0.90)×10^-3^	33.14 (9.19-66.35)×10^3^
Metastases (n=21)	12.00 (0.00-20.70) ^▴^	0.37 (0.24-0.57)×10^-3♦^	35.40 (0.00-66.35)×10^3•^
No metastasis (n=23)	7.80 (3.30-23.48)	0.49 (0.28-0.96)×10^-3^	14.77 (5.96-43.38)×10^3^

^99m^Tc-PSMA SPECT/CT, ^99m^Tc-labelled prostate-specific membrane antigen molecular probe single photon emission computed tomography; mpMRI, multiparametric magnetic resonance imaging.

Comparison of SUV_max_ between groups: ^△^compared to the prostate cancer group, P=0.005; ^▴^compared with the no metastasis subgroup, P=0.007; ^*^ compared between the Gleason score subgroup, P < 0.001.

Comparison of ADC_min_ between groups: ^□^compared to the prostate cancer group, P=0.002; ^♦^compared with the no metastasis subgroup, P=0.009; ^#^ compared between the Gleason score subgroup, P=0.012.

Comparison of SUV_max_/ADC_min_ between groups: ^▽^compared to the prostate cancer group, P=0.003; ^•^compared with the no metastasis subgroup, P=0.003; ^★^ compared between the Gleason score subgroup, P < 0.001.

**Table 4:** Multivariate logistic regression analysis of related factors of Gleason score.

Independent variable	OR value	95% CI for OR value		*P-*value
		Lower	Upper	
Age (years)	0.998	0.864	1.152	0.977
PSA (ng/mL)	1.028	0.977	1.081	0.288
SUV_max_/ADC_min_ (×10^3^)	1.557	1.015	2.388	0.042

CI, confidence interval; OR, odds ratio; PSA, prostate specific antigen, SUV_max_/ADC_min_, maximum standardized uptake value/minimum apparent diffusion coefficient.

## Discussion

The labeling method for ^99m^Tc-HYNIC-Glu-Urea-A(^99m^Tc-PSMA) is simple and has high radiochemical purity (10). Previous studies have demonstrated the high diagnostic efficacy of ^99m^Tc-PSMA SPECT/CT in detecting recurrent biochemical lesions after radical prostatectomy and bone metastases of PCa (9, 20). With the introduction of imaging technology, the diagnosis and initial management of localized PCa are increasingly dependent on imaging findings. ^99m^Tc-PSMA SPECT/CT is predominantly used in the primary PCa staging of regional and distant diseases. However, little is known about the value of ^99m^Tc-PSMA SPECT/CT for the primary detection of lesions within the prostate. To our knowledge, this is the first comparison between ^99m^Tc-PSMA SPECT/CT and mpMRI for primary PCa lesions.

**Figure 6:**
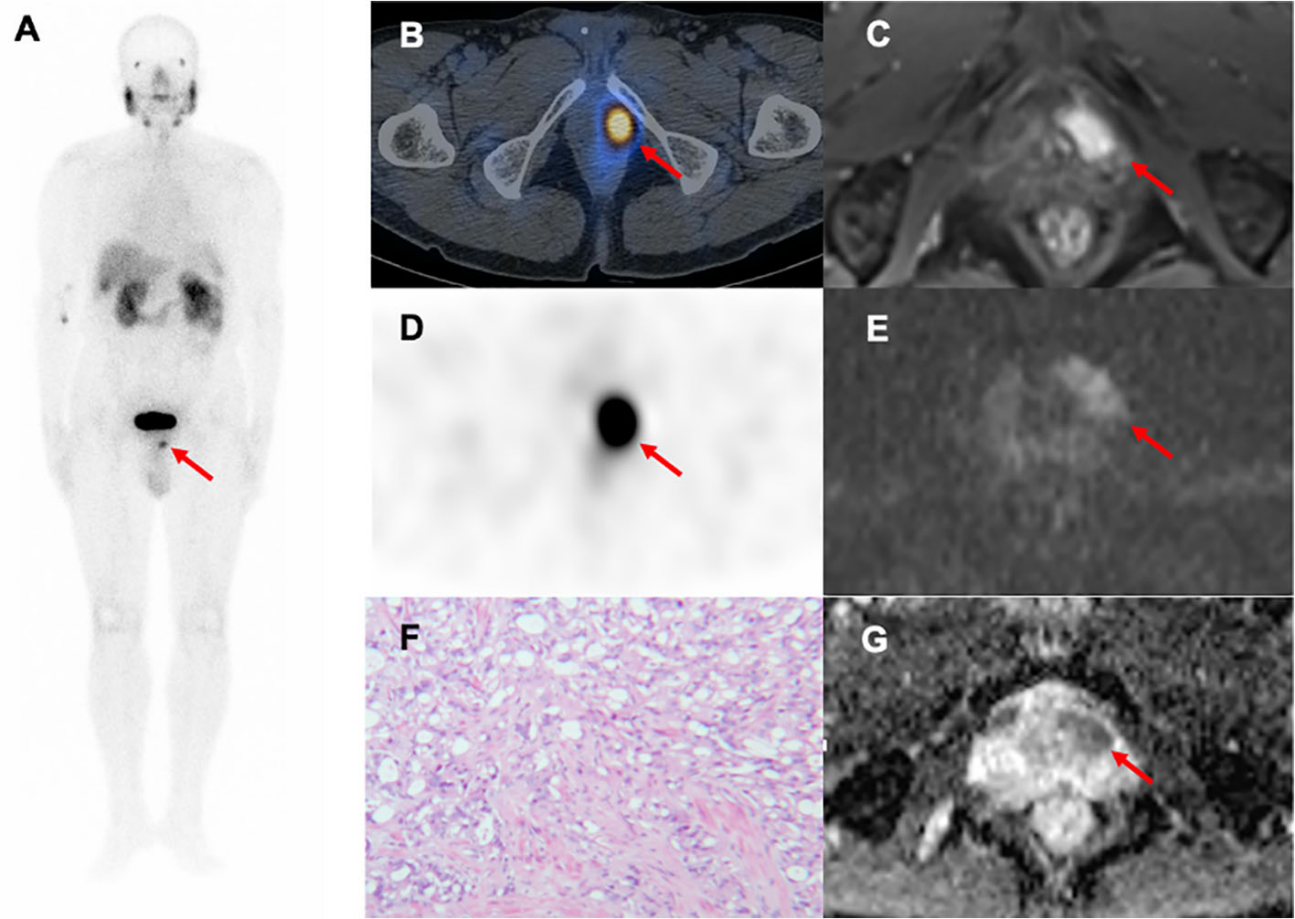
A 79-year-old man with progressive dysuria. The PSA level was 15.31 ng/mL at the time of ^99m^Tc-PSMA SPECT/CT and mpMRI. Whole-body planar ^99m^Tc-PSMA **(A)** and transverse SPECT/CT **(B, D)** showed foci of increased PSMA uptake in the left-anterior (red arrow, SUV_max_=13.10, SUV_max_/ADC_min_=16.7×10^3^). Hypointense signals were shown on apparent diffusion coefficient (ADC) **(G)**, red arrow), and hyperintense signals on diffusion-weighted imaging (DWI) **(E)**, red arrow) in the left transitional band of the prostatic apex. Dynamic contrast-enhanced (DCE) scanning was significantly enhanced **(C)**, red arrow). **(F)** Prostatic lesion was confirmed pathologically as prostate cancer (hematoxylin and eosin (HE) staining, 100×magnification; Gleason score: 4 + 5 = 9). ^99m^Tc-PSMA SPECT/CT, ^99m^Tc-labelled prostate-specific membrane antigen molecular probe single photon emission computed tomography; mpMRI, multiparametric magnetic resonance imaging; SUV_max_, maximum standardized uptake value; ADC_min_, the minimum apparent diffusion coefficient.

In our cohort study, ^99m^Tc-PSMA SPECT/CT and mpMRI had limited specificity for detecting primary PCa lesions. SUV_max_ and ADC_min_ are important quantitative parameters in SPECT/CT and mpMRI, respectively. Typical PCa foci showed localized high-uptake foci on ^99m^Tc-PSMA SPECT/CT and low-signal foci on mpMRI ADC maps (**Figure 6**). Previous studies on PCa detection by PET have shown that there may be a certain degree of correlation between mpMRI and PET parameters in the same PCa lesion; that is, SUV_max_ and ADC_min_ were negatively correlated (21, 22). Therefore, it is essential to study whether combining these two imaging techniques can further improve the diagnostic efficacy of PCa. In our study, SUV_max_ was positively correlated with the Gleason score, while ADC_min_ was negatively correlated. Based on the above results, we combined the two parameters and used the ratio to construct a new parameter (SUV_max_/ADC_min_) to obtain a more significant correlation with the Gleason score. The results of this study are consistent with our expectations. SUV_max_/ADC_min_ was positively correlated with the Gleason score, which was also consistent with previous ^68^Ga-PSMA PET/CT and mpMRI-related research results (23–25). In addition, Schmidkonz et al. confirmed that SUV_max_ in prostatic lesions could be used to predict primary PCa and lymph node and bone metastases. This may be the higher the uptake of PSMA in prostatic lesions, the higher the malignancy of the lesions, resulting in an increased risk of bone or lymph node metastasis (25). However, approximately 10% of patients with primary PCa have low PSMA expression (26). Some false-negative results were obtained in the clinical setting when SUV_max_ was used separately. The ratio SUV_max_/ADC_min_ synthesizes the expression of PSMA and the degree of diffusion of water molecules (27). The present study found that the diagnostic efficacy of SUV_max_/ADC_min_ was better than that of SUV_max_ or ADC_min_ alone. SUV_max_/ADC_min_ may be used as a predictive parameter for PCa, helping to distinguish benign and malignant lesions of PCa and determine whether there were metastases. The result was also consistent with previous ^18^F-choline PET/MRI related research (21). Zhang et al. conducted a retrospective ^68^Ga-PSMA-11 PET/CT analysis of 42 patients with moderate-and high-risk PCa who underwent RP, and found that SUV_max_ in local prostate lesions was significantly higher in the group with pelvic lymph node metastases than in the group without lymph node metastases (28). In our study, the larger the prostatic lesion size, the higher the SUV_max_/ADC_min._ When SUV_max_/ADC_min_ in the prostatic lesion was >7.0×10^3^, the lesion was more likely to be malignant. When SUV_max_/ADC_min_ in the prostatic lesion is >27.0×10^3^, the patient with PCa may have lymph node and bone metastases. Hence, we postulated that SUV_max_ combined with ADC_min_ (SUV_max_/ADC_min_) might decrease bias and improve diagnostic accuracy.

The prognosis of PCa is closely related to the Gleason score grading system (29). The Gleason score is a critical indicator of the pathological results of prostate biopsy. In the previous study on PSMA, Kasperzyk et al. evaluated the expression of PSMA in PCa tissues by immunohistochemical staining, and found that Gleason score in the group with high PSMA expression was significantly higher than that in the group with low PSMA expression (30). Uprimny et al. retrospectively analyzed the ^68^Ga-PSMA-11 PET/CT examination data of 90 patients with PCa confirmed by prostate biopsy, and found that SUV_max_ was significantly positively correlated with Gleason score (31).In our study, patients with PCa were divided into high-,medium- and low-risk groups with a Gleason score of 7 as the cut-off value. Our results showed that SUV_max_/ADC_min_ was the main predictor of the high-risk group, with an optimal cut-off value of 15.0×10^3^. This suggests that SUV_max_/ADC_min_ ratio is a useful imaging parameter for evaluating tumor biology and prognosis, which may significantly impact the selection of therapeutic strategies.

This study had some limitations. Among the 44 patients diagnosed with PCa, 21 did not undergo RP, and gross specimens could not be obtained; only puncture biopsy could be used as the final pathological result. The pathological grading of puncture lesions may differ from actual grading. Furthermore, this was a single-center study with a small sample size, and the conclusions should be verified in a large-scale sample cohort. We did not evaluate the role of SUV_max_/ADC_min_ in predicting prognosis at follow-up. However, this exploratory study is still valuable as the first clinical quantitative application of ^99m^Tc-PSMA SPECT/CT combined with mpMRI in PCa lesions. In a future study, we aim to develop a novel analytical approach based on a radiomics quantitative model derived from ^99m^Tc-PSMA SPECT/CT and mpMRI for noninvasive prediction of intraprostatic lesions in patients with PCa and prognosis.

## Conclusion

In this prospective study, our results revealed that combined ^99m^Tc-PSMA SPECT/CT and mpMRI had a higher diagnostic accuracy for detecting treatment-naive PCa than either modality alone. In addition, SUV_max_/ADC_min_ is a promising molecular imaging parameter for diagnosing PCa and evaluating its biological behavior.

## Data availability statement

The raw data supporting the conclusions of this article will be made available by the authors, without undue reservation.

## Ethics statement

The studies involving humans were approved by the ethics committee of Fujian Provincial Hospital. The studies were conducted in accordance with the local legislation and institutional requirements. The participants provided their written informed consent to participate in this study. Written informed consent was obtained from the individual(s) for the publication of any potentially identifiable images or data included in this article.

## Author contributions

Conceptualization: WC. Data curation: YZ. Formal analysis: YZ. Investigation: YZ. Methodology: YZ. Project administration: WC and ZL. Resources: WC, TL, YW, and LY. Software: YZ and YS. Supervision: WC and ZL. Validation: WC and ZL. Visualization: YZ and YS. Roles/Writing-original draft: YZ. Writing- review & editing: WC. All authors contributed to the article and approved the submitted version.
